# Increased Frequency of Peripheral B and T Cells Expressing Granulocyte Monocyte Colony-Stimulating Factor in Rheumatoid Arthritis Patients

**DOI:** 10.3389/fimmu.2017.01967

**Published:** 2018-01-10

**Authors:** Anastasia Makris, Sofia Adamidi, Christos Koutsianas, Christina Tsalapaki, Emilia Hadziyannis, Dimitrios Vassilopoulos

**Affiliations:** ^1^Joint Rheumatology Program, Clinical Immunology-Rheumatology Unit, 2nd Department of Medicine and Laboratory, School of Medicine, National and Kapodistrian University of Athens, Hippokration General Hospital, Athens, Greece

**Keywords:** rheumatoid arthritis, granulocyte monocyte colony-stimulating factor, methotrexate, tumor necrosis factor inhibitors, cytokines

## Abstract

**Objectives:**

Granulocyte monocyte colony-stimulating factor (GM-CSF) is currently considered a crucial inflammatory mediator and a novel therapeutic target in rheumatoid arthritis (RA), despite the fact that its precise cellular sources remain uncertain. We studied the expression of GM-CSF in peripheral lymphocytes from RA patients and its change with antirheumatic therapies.

**Methods:**

Intracellular GM-CSF expression was assessed by flow cytometry in stimulated peripheral B (CD19+) and T (CD3+) cells from RA patients (*n* = 40), disease (*n* = 31 including osteoarthritis *n* = 15, psoriatic arthritis *n* = 10, and systemic rheumatic diseases *n* = 6) and healthy (*n* = 16) controls. The phenotype of GM-CSF+ B cells was assessed as well as longitudinal changes in GM-CSF+ lymphocytes during methotrexate (MTX, *n* = 10) or anti-tumor necrosis factor (anti-TNF, *n* = 10) therapy.

**Results:**

Among untreated RA patients with active disease (Disease Activity Score 28-C-reactive protein = 5.6 ± 0.89) an expanded population of peripheral GM-CSF+ B (4.1 ± 2.2%) and T (3.4 ± 1.6%) cells was detected compared with both disease (1.7 ± 0.9%, *p* < 0.0001 and 1.7 ± 1.3%, *p* < 0.0001, respectively) and healthy (0.3 ± 0.2%, *p* < 0.0001 and 0.6 ± 0.6%, *p* < 0.0001) controls. RA GM-CSF+ B cells displayed more commonly a plasmablast or transitional phenotype (37.12 ± 18.34% vs. 14.26 ± 9.46%, *p* = 0.001 and 30.49 ± 15.04% vs. 2.45 ± 1.84%, *p* < 0.0001, respectively) and less a memory phenotype (21.46 ± 20.71% vs. 66.99 ± 16.63%, *p* < 0.0001) compared to GM-CSF− cells. GM-CSF expression in RA patients did not correlate to disease duration, activity or serological status. Anti-TNF treatment led to a statistically significant decrease in GM-CSF+ B and T cells while MTX had no significant effect.

**Discussion:**

This is the first study showing an expanded population of GM-CSF+ B and T lymphocytes in patients with active RA which declined after anti-TNF therapy.

## Introduction

Rheumatoid arthritis (RA) is a chronic autoimmune disease associated with joint and systemic inflammation and autoantibody production [rheumatoid factor (RF) and anticyclic citrullinated peptide (anti-CCP) antibodies], which untreated leads to cartilage and bone destruction as well as to systemic complications (1). Its pathogenesis is multifactorial involving the contribution of certain genetic and environmental factors. An expanded range of immune cells recruited from the circulation (including B and T lymphocytes, natural killer cells, γδ T cells, myeloid cells, mast cells) as well as resident (synovial fibroblasts, macrophage-like cells) cells through the secretion of various cytokines [such as tumor necrosis factor (TNF), interleukin-1 (IL-1), IL-6, and IL-17A] contribute to joint and systemic inflammation that characterizes the disease and which untreated leads to chronic joint damage (2).

Over the past 15 years, biologic disease modifying antirheumatic drugs (bDMARDs or biologics) targeting specific immune cells (B cells: rituximab) or their interaction (antigen-presenting cells/T cells: abatacept) as well as proinflammatory cytokines (TNF: anti-TNF agents, IL-6: tocilizumab, IL-1: anakinra) have been developed and successfully introduced in clinical practice, confirming the important role of these therapeutic targets in disease pathogenesis (1).

Granulocyte macrophage colony-stimulating factor (GM-CSF) has emerged as a critical inflammatory mediator in RA and recently, as a novel therapeutic target (3, 4). GM-CSF is elevated in the serum (5) and synovial fluid (6, 7) of RA patients, is expressed in rheumatoid synovium (8), its *in vivo* administration can induce RA exacerbation (9) while its inhibition has beneficial effects in animal models of arthritis (10) and patients with RA (11–14). Furthermore, it appears that GM-CSF is also involved in the generation of inflammatory and arthritic joint pain (15).

Although numerous cells have been reported to produce GM-CSF (including macrophages, activated T and B cells, synovial fibroblasts, endothelial cells, etc.) (3), the specific cellular sources of GM-CSF in RA patients are unknown. Recently, a subset of B cells producing GM-CSF called innate response activator (IRA) B cells have been recognized and are thought to participate in innate immunity responses (16). Whether such a subset of GM-CSF producing B cells exists in human RA patients is unknown.

The aim of our study was to determine whether peripheral T or B lymphocytes producing GM-CSF exist in RA patients as well as in disease or healthy controls and how they are being modified with current antirheumatic therapies.

## Materials and Methods

### Study Population

Forty untreated patients with RA, 31 disease controls (osteoarthritis *n* = 15, psoriatic arthritis-PsA *n* = 10 and systemic rheumatic diseases *n* = 6: Sjögren’s syndrome *n* = 2, antineutrophil cytoplasmic antibody-ANCA-associated vasculitis *n* = 2, myositis *n* = 1, giant cell arteritis *n* = 1) followed at our Unit (Clinical Immunology-Rheumatology Unit, 2nd Department of Medicine and Laboratory, Hippokration General Hospital, Athens, Greece), as well as 16 healthy controls were included in the study. All RA patients fulfilled the 2010 American College of Rheumatology/European League against Rheumatism (ACR/EULAR) classification criteria (17).

All RA or disease control patients who were receiving corticosteroids, conventional synthetic DMARDs (csDMARDs) or bDMARDs at the time of study enrollment were excluded. Patients with active infection, malignancies, or other coexisting autoimmune or inflammatory diseases were also excluded. This study was carried out in accordance with the recommendations of the Declaration of Helsinki with written informed consent from all subjects. The protocol was approved by the hospital’s scientific committee (Hippokration General Hospital, Athens, Greece).

For each patient demographic characteristics, laboratory tests for erythrocyte sedimentation rate, C-reactive protein (CRP), RF and anti-CCP antibodies, disease activity [assessed by the Disease Activity Score (DAS)-28-CRP] and function [assessed by the Health Assessment Questionnaire (HAQ) score] indices were determined at the day of sample collection. A subgroup of patients who subsequently started therapy with methotrexate (MTX) or anti-TNF agents were followed longitudinally and the same evaluation was repeated after a median time of 3 months.

### Antibodies and Reagents

Fetal bovine serum (FBS), RPMI 1640 culture medium, phosphate-buffered saline (PBS), and antibiotic solution (penicillin/streptomycin) were purchased from GIBCO (Gibco BRL Laboratories, Grand Island, NY, USA). Ficoll-Histopaque 1077-1, phorbol 12-myristate 13-acetate (PMA), and brefeldin-A (BFA) were purchased from SIGMA (St. Louis, MO, USA), Ionomycin from ENZO Life Sciences (USA), 7-AAD viability staining solution and reagents for cell staining, fixation and permeabilization as well all monoclonal antibodies used for flow cytometric analysis such as: anti-CD19-fluorescein isothiocyanate (FITC), anti-GM-CSF-phycoerythrin (PE), anti-CD3-FITC, anti-CD27-allophycocyanin (APC), anti-CD38-PE/Cy5 and appropriate isotype controls mouse IgG1-FITC, Rat IgG2α-PE, mouse IgG1-APC, and mouse IgG1-PE/Cy5 were obtained from Biolegend (CA, USA). Finally, the 96 rounded bottom well plates were purchased from Costar (Cambridge, MA, USA).

### Cell Surface and Intracellular Cytokine Staining Assays

Heparinized blood was drawn from patients and controls and peripheral blood mononuclear cells (PBMCs) were isolated by Ficoll—Histopaque density gradient centrifugation at room temperature. Cells were then washed twice with PBS and viability was estimated by trypan blue dye exclusion. The cells were then resuspended at 2 × 10^6^ cells/mL in RPMI 1640 medium supplemented with 10% FBS (heat inactivated to 56°C for 1 h), 2 mM l-glutamine, 100 U/mL penicillin, and 100 µg/mL streptomycin and were plated in 96-well plates. Cells were cultured in the presence of medium alone or PMA (25 ng/mL) and ionomycin (1 µg/mL) for 16 h at 37°C in an atmosphere of 95% air and 5% CO_2_. To inhibit cytokine secretion, BFA (10 µg/mL) was added 1 h after starting incubation.

Following overnight incubation, cells were collected and washed once in ice-cold PBS, and were then stained with surface antibodies against CD19 (anti-CD19-FITC), CD3 (anti-CD3-FITC), CD27 (anti-CD27-APC), CD38 (anti-CD38-PE/Cy5), or appropriate isotype controls for 20 min in the dark in cell staining buffer (PBS/5% FBS). Cells were then washed, fixed and permeabilized according to the manufacturer’s instructions (Biolegend). After permeabilization, cells were stained with either antihuman GM-CSF-PE or its isotype control (IgG2α-PE) for 20 min at RT in the dark. Cells were then washed in permeabilization buffer and resuspended in cell staining buffer. Stained cells were analyzed using FACS flow cytometry (PARTEC). Dead cells were identified and excluded from all analysis by 7AAD according to the manufacturer’s instructions. In certain experiments, the efficiency of intracellular staining was tested using staining for interferon-γ (IFN-γ) as a positive control after stimulation with PMA and ionomycin as described above (data not shown).

### Statistical Analyses

Percentages of cells expressing cell surface markers and cytokine-producing cells were described as mean ± SD for each individual in each group. The nonparametric Mann–Whitney rank-sum test was used for comparison between groups. For multiple comparisons between groups the Bonferroni correction was applied. Paired data for patients before and after treatment were compared using a paired Wilcoxon matched signed rank sum test. The SPSS statistical package version 20.0 was used for analysis while for all test results *p*-values of <0.05 were considered statistically significant.

## Results

### Clinical and Demographic Characteristics

The characteristics of the 40 RA patients included in the study are shown in Table 1. The majority of patients were females (83%) with a mean age of 60.9 ± 11.7 years (range: 36–80 years). All RA patients had active disease (mean DAS-28-CRP = 5.6 ± 0.9, median = 5.6) with a median disease duration of 15 months while none of them was receiving any immunosuppressive treatment including corticosteroids at the time of inclusion in the study. Sixteen healthy (12 females/4 males of similar age) and 31 disease controls (23 female/8 males of similar age) were also included in the study (see Materials and Methods).

**Table 1:** Baseline clinical and demographic characteristics of rheumatoid arthritis (RA) patients.

Characteristic	
*n*	40
Age (years)	60.9 ± 11.7
Females, *n* (%)	33 (83%)
Disease duration (months)	25.3 ± 28.1 (15)
DAS-28 (CRP)	5.6 ± 0.9 (5.6)
HAQ	1.0 ± 0.6 (1.1)
Swollen joint count (SJC)	13.6 ± 5.4 (13)
Tender joint count (TJC)	13.6 ± 6.1 (13)
CRP (mg/L)	19.9 ± 18.7 (13.9)
Rheumatoid factor+, *n* (%)	20 (50)
Anti-CCP+, *n* (%)	14 (35)

*Data are expressed as mean ± 1 SD (median in parenthesis), unless otherwise specified*.

*DAS-28 (CRP), Disease Activity Score in 28 joints; CRP, C-reactive protein; RF, rheumatoid factor; Anti-CCP, anticyclic citrullinated peptide antibodies; HAQ, Health Assessment Questionnaire*.

### Peripheral B and T Cells

**Figure 1:**
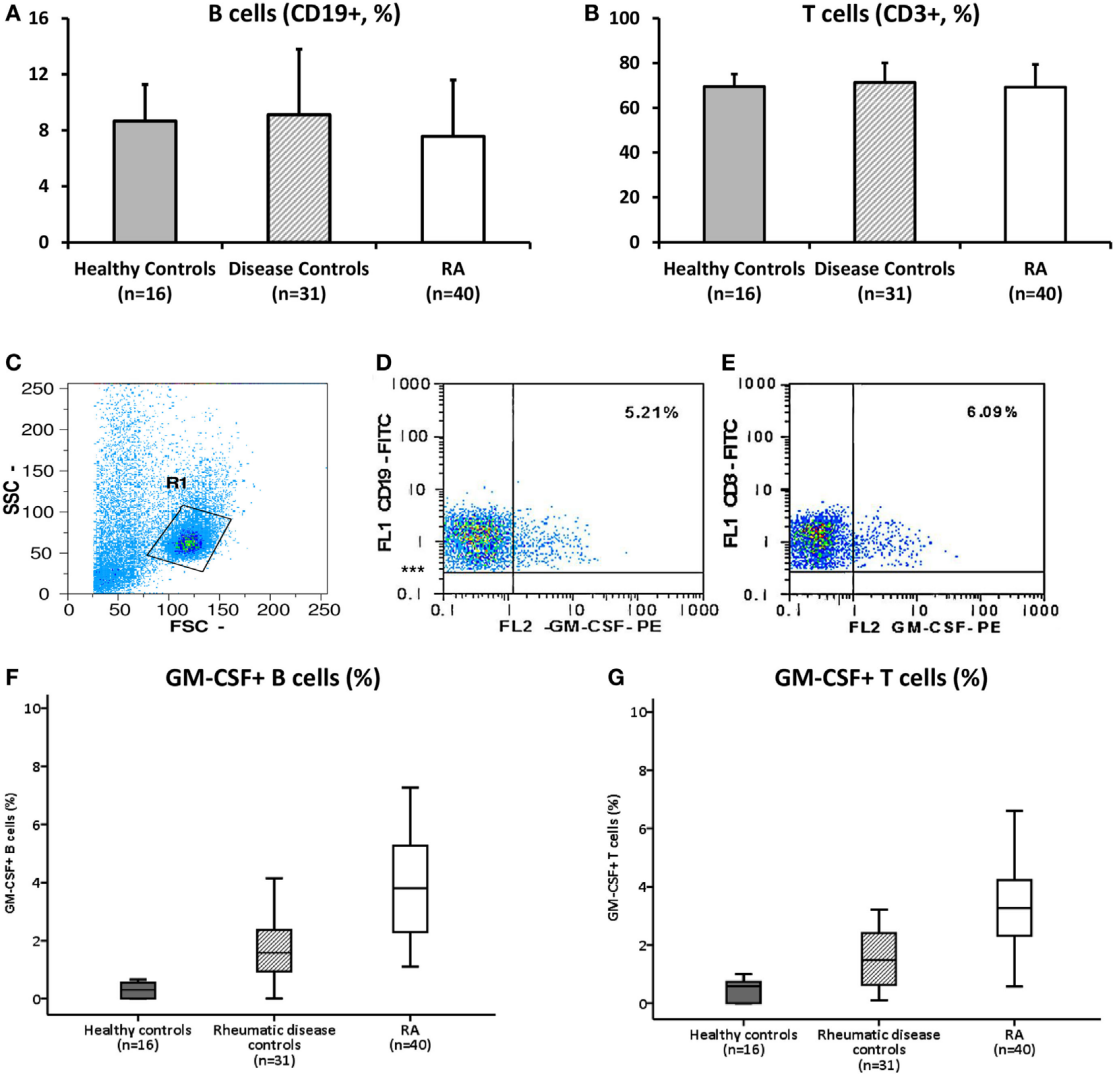
Granulocyte monocyte colony-stimulating factor (GM-CSF) expression in peripheral B and T cells from RA patients and controls. **(A,B)**. Flow cytometric analysis of B [CD19+, **(A)**] and T [CD3+, **(B)**] cells of healthy controls (grey bars, *n* = 16), disease controls (diagonal bars, *n* = 31), and rheumatoid arthritis (RA, white bars, *n* = 40) patients is shown. Bars represent the mean ± 1 SD % of CD19+ and CD3+ cells. No statistically significant difference between groups was found. **(C–E)**. Representative flow cytometric dot-plots showing the population of B and T cells expressing GM-CSF in a patient with RA. After initial gating on lymphocytes [Gate R1, **(C)**], based on their SSC and FSC properties, the expression of GM-CSF+ cells among B [CD19+, **(D)**] or T [CD3+, **(E)**] gated cells was calculated. **(F,G)**. The respective % of GM-CSF+ B [CD19+, **(F)]** and T [CD3+, **(G)**] cells in healthy controls (white bars, *n* = 16), disease controls (diagonal bars, *n* = 31), and RA (white bars, *n* = 40) is shown as box-plots. *p* < 0.0001 for all comparisons between groups (see text for details).

There was no statistically significant difference in the % of peripheral blood B (CD19+, RA: 7.6 ± 4.0% vs. disease controls: 9.1 ± 4.7% vs. healthy controls: 8.7 ± 2.6%, *p* = NS) or T cells (CD3+, RA: 69.2 ± 10.1% vs. disease controls: 71.3 ± 8.8% vs. healthy controls: 69.5 ± 5.6%, *p* = NS) between RA patients and controls (Figures 1A,B).

### GM-CSF Expression in Peripheral B and T Cells

There was no expression of GM-CSF in resting peripheral lymphocytes (data not shown) but a distinct cell subpopulation was evident in PBMCs stimulated overnight with PMA and ionomycin. A representative flow cytometric dot-plot showing the intracellular GM-CSF expression among the B and T cell gated subpopulation in a patient with RA is shown in Figures 1C–E.

As shown in Figure 1F, the percentage of peripheral B cells (CD19+) GM-CSF+ cells was significantly higher in RA patients (4.1 ± 2.2%, *n* = 40) compared to disease (1.7 ± 0.9%, *n* = 31, *p* < 0.0001) and healthy (0.3 ± 0.2%, *n* = 16, *p* < 0.0001) controls. Among disease controls, patients with osteoarthritis (*n* = 15) displayed a higher percentage of GM-CSF+ B cells (2.2 ± 1.0%) compared to patients with a non-RA inflammatory arthritis (PsA, *n* = 10, 0.9 ± 0.2%) or systemic inflammatory rheumatic diseases (*n* = 8, 1.5 ± 0.7%). When the comparison in the expression of GM-CSF in B cells from RA patients was restricted only to patients with osteoarthritis or PsA the difference remained statistically significant (*p* = 0.002 and *p* < 0.0001, respectively). The difference between RA and the different patient subgroups and healthy controls remained significant after the Bonferroni correction for multiple comparisons (data not shown).

Similarly to B cells, an expanded T cell (CD3+) population expressing GM-CSF was seen in RA patients (3.4 ± 1.6%, *n* = 40) compared with both disease (1.7 ± 1.3%, *n* = 31, *p* < 0.0001) and healthy (0.6 ± 0.6%, *n* = 16, *p* < 0.0001) controls (Figure 1G). Among disease controls, patients with systemic rheumatic diseases (*n* = 6) displayed a higher percentage of GM-CSF+ T cells (2.5 ± 2.3%) compared to patients with osteoarthritis (1.8 ± 1.0%) or PsA (1.0 ± 0.6%). Still the difference in T cell GM-CSF expression between patients with RA and osteoarthritis or PsA was statistically significant (*p* = 0.001 and *p* < 0.0001, respectively). Similarly to the B cell GM-CSF expression, the difference remained significant after the Bonferroni correction for multiple comparisons, with the exception in the comparison between the RA and systemic rheumatic disease group (*p* = 0.924, rest of data not shown).

### Phenotype of GM-CSF+ vs. − B Cells

**Figure 2:**
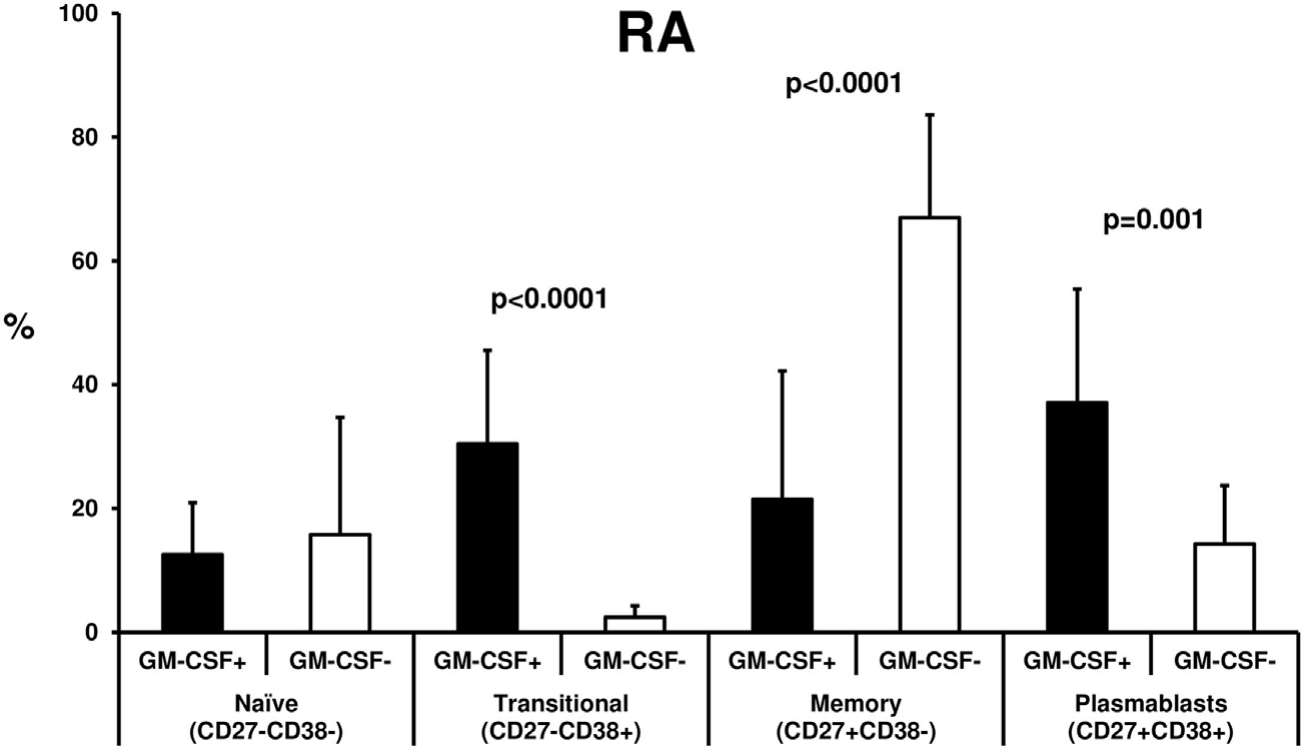
B cell phenotype of rheumatoid arthritis (RA) granulocyte monocyte colony-stimulating factor (GM-CSF+) and − cells. Freshly isolated PBMCs from 15 RA patients were stimulated with PMA and inonomycin and then stained with specific antibodies against CD19 (anti-CD19-FITC), GM-CSF (anti-GM-CSF-PE), CD27 (anti-CD27-APC), and CD38 (anti-CD38-PE/Cy5, as described in Materials and Methods). B cells (CD19+) either GM-CSF+ or − were classified as: transitional (CD38+ CD27−), memory (CD27+ CD38−), plasmablasts (CD27+ CD38+), or naive B cells (CD27− CD38−). The % of each B cell subpopulation was compared between GM-CSF+ (black bars) and − (white bars) cells. The bars represent the mean ± SD (%) of each subtype. The statistically significant *p* values (<0.05) from the comparison between the two groups are shown.

In 15 patients with RA, as well as in 8 disease controls (osteoarthritis *n* = 4, PsA *n* = 4) the phenotype of B cells (either GM-CSF+ or −) was assessed by flow cytometry (Figure 2). CD19+ gated cells were classified as: transitional (CD27− CD38+), memory (CD27+ CD38−), plasmablasts (CD27+ CD38+), and naive (CD27− CD38−) after staining for the CD27 and CD38 surface markers. A comparative analysis between GM-CSF+ and − cells, showed an increased plasmablast (37.12 ± 18.34%) and transitional (30.49 ± 15.04%) cell population among GM-CSF+ compared to GM-CSF− (14.26 ± 9.46%, *p* = 0.001 and 2.45 ± 1.84%, *p* < 0.0001, respectively) in RA B cells (Figure 2). In contrast, the percentage of memory cells was lower in GM-CSF+ cells (21.46 ± 20.71%) compared to GM-CSF− cells (66.99 ± 16.63%, *p* < 0.0001).

A similar pattern was also observed in the disease controls (see Figure S1 in Supplementary Material) with the exception of naive B cells which were higher in GM-CSF+ B cells. In healthy controls due to the small percentage of GM-CSF+ B cells such analysis was not possible.

### GM-CSF Expression According to RA Disease Activity or Duration and Serological Status

The high expression of GM-CSF in peripheral B and T lymphocytes could be related to the disease activity of the included RA patients. We did not find any correlation between B or T cell GM-CSF expression and serum CRP levels, disease activity (measured by the DAS-28-CRP score), or disease duration (data not shown). There was also no difference in GM-CSF expression between RF/anti-CCP+ vs. RF/anti-CCP− RA patients (Figure S2 in Supplementary Material).

### Effect of Antirheumatic Therapies on GM-CSF Expressing T or B Cells

Whether antirheumatic therapy (either non-biologic or biologic) could influence the number of GM-CSF expressing T or B lymphocytes from RA patients is currently unknown.

**Figure 3:**
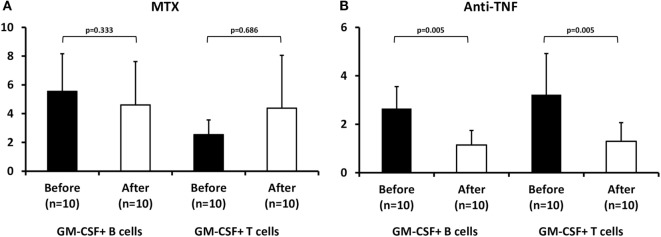
Effect of methotrexate (MTX) and antitumor necrosis factor (anti-TNF) treatment on granulocyte monocyte colony-stimulating factor (GM-CSF) expression in B and T cells from rheumatoid arthritis (RA) patients. Flow cytometric analysis of GM-CSF+ B (CD19+) and T (CD3+) of RA patients before (baseline, black bars) and after 3 months of therapy (white bars) with **(A)** MTX (*p* = NS) or **(B)** anti-TNF agents+ MTX (*n* = 10, *p* = 0.005).

We first tested the effect of MTX in 10 treatment-naive RA patients after a median 3-month treatment period. We noted a non-significant decrease in GM-CSF expressing B cells during MTX treatment (from 5.5 ± 2.6 to 4.6 ± 3.0%, *n* = 10, *p* = 0.333, Figure 3A) whereas the opposite was seen in GM-CSF expressing T cells (a non-significant increase from 2.5 ± 1.0 to 4.4 ± 3.7%, data available from nine patients, *p* = 0.686, Figure 3A). This pattern of response was similar in responders (*n* = 5) or non-responders (*n* = 5) to therapy (data not shown).

In contrast, in 10 RA patients who had failed MTX treatment and an anti-TNF agent (etanercept *n* = 4, adalimumab *n* = 3, certolizumab pegol *n* = 2, golimumab *n* = 1) was added to MTX, both B and T cells expressing GM-CSF declined significantly during treatment (B cells: from 2.65 ± 0.9 to 1.14 ± 0.6%, *p* = 0.005, T cells: from 3.23 ± 1.7 to 1.3 ± 0.8%, *p* = 0.005, Figure 3B). This decrease was more pronounced in responders (*n* = 6, B cells: from 3.15 ± 0.82 to 1.33 ± 0.63%, *p* = 0.01, T cells: from 3.65 ± 2.0 to 1.37 ± 0.71%, *p* = 0.028) than in non-responders (*n* = 4, B cells: from 1.9 ± 0.3 to 0.9 ± 0.5%, *p* = 0.068, T cells: from 2.6 ± 1.0 to 1.2 ± 1.0%, *p* = 0.068) to anti-TNF therapy.

## Discussion

This is the first study in the literature demonstrating an expanded population of peripheral B and T lymphocytes expressing GM-CSF in treatment-naive patients with active RA. Peripheral B cells expressing GM-CSF had more commonly a plasmablast and transitional phenotype compared to GM-CSF-negative cells. Biologic treatment with anti-TNF agents led to a statistically significant decrease in both B and T cells expressing GM-CSF while non-biologic treatment with MTX therapy had contrasting results (increased GM-CSF+ T and decreased GM-CSF+ B cells).

GM-CSF apart from its role as a hematopoietic growth factor, it has been also recognized as a significant mediator in various inflammatory diseases including arthritis (10, 18). Previous studies have shown that GM-CSF is elevated in the synovial fluid (6, 7, 19) or serum (5) of patients with RA while GM-CSF expressing cells have been found in the synovium (8) or synovial fluid (6, 20–23) of these patients. Although a number of cell types have been reported to secrete GM-CSF such as macrophages, dendritic cells, activated T cells, and synovial fibroblasts (3), there are limited data for its source in RA patients.

Previous studies in animal models of experimental autoimmune encephalomyelitis (24) and myocarditis (25) have shown that CD4+ T cells secreting GM-CSF were critical in their pathogenesis. Recently, Piper et al. also reported an expanded population of GM-CSF secreting CD4+ T cells in the synovial fluid of patients with juvenile idiopathic arthritis (21). In our study, we found an expanded population of peripheral T cells expressing GM-CSF in untreated patients with active RA compared to disease controls with inflammatory (PsA) or non-inflammatory (osteoarthritis) arthritis as well as to patients with systemic rheumatic diseases (vasculitis, Sjogren’s syndrome, myositis). This peripheral T cell subpopulation did not change significantly with MTX treatment (a non-significant increase was noted) while the anti-TNF therapy led to a statistically significant decrease of GM-CSF+ T cells.

Although T cells producing GM-CSF have been previously described in RA, this is the first study showing the existence of a peripheral B cell subpopulation expressing GM-CSF in RA patients. Rauch et al. have recently identified a unique B cell subpopulation secreting GM-CSF in the spleen of mice and humans in a sepsis model (16); these B cells referred as IRA B cells participate in innate immune responses and have been found also to promote local IgM production during lung infections (26). More recently, B cells expressing GM-CSF have been detected in patients with multiple sclerosis and have been shown to activate efficiently myeloid cells (27).

In our study, peripheral GM-CSF B cell expression was much higher in RA patients compared to disease controls with systemic rheumatic diseases such as ANCA vasculitides and Sjögren syndrome, diseases where B cells are crucial to their pathogenesis. These findings could indicate a specific pathogenetic role of this B cell subpopulation in RA.

We did not find any correlation between GM-CSF T or B cell expression and disease activity, although the majority of our patients had high disease activity since they were not receiving any antirheumatic therapies at the time of inclusion in the study.

Similarly to T cells, non-biologic (MTX) treatment led to a non-significant change in this B cell subpopulation (small decrease), whereas biologic (anti-TNF) therapy led to a statistically significant decrease. In anti-TNF treated patients, this decrease was more pronounced in responders to therapy, although the number of studied patients was small for any definite conclusions to be made (data not shown).

Phenotypic analysis revealed that GM-CSF expressing B cells demonstrated more commonly a plasmablast and transitional phenotype compared to GM-CSF− cells. Plasmablasts are generated from recently activated naive and memory B cells during immune responses (28) and has been recently shown to produce autoantibodies that target various autoantigens in RA including α-enolase, citrullinated histone H2B, and citrullinated fibrinogen (29). Although the function of these circulating GM-CSF expressing plasmablasts and transitional cells is currently unknown, they could participate in autoantibody production and tissue damage in RA patients. This was not though a unique feature of RA GM-CSF+ B cells since a similar pattern was observed in GM-CSF+ B cells from patients with PsA and osteoarthritis.

In view of the recent findings that specific inhibition of GM-CSF has beneficial effects in RA patients (11–14), our study identified a unique circulating population of GM-CSF secreting peripheral T and B lymphocytes that could participate in the inflammatory process that characterizes RA. Although the number of included patients was small, the findings were consistent in RA patients compared with both disease and healthy controls.

In conclusion, our study shows for the first time a unique subpopulation of peripheral GM-CSF secreting cells among T and particularly B cells from patients with active RA that is vulnerable to anti-TNF therapy. More studies elucidating the precise role of these cells in innate or adaptive immune responses in RA are needed.

## Ethics Statement

This study was carried out in accordance with the recommendations of the Declaration of Helsinki with written informed consent from all subjects. The protocol was approved by the hospital’s scientific committee (Hippokration General Hospital, Athens, Greece).

## Author Contributions

Design of the study, data analysis, and manuscript preparation: AM, SA, and DV. Acquisition of data: AM, SA, CK, CT, EH, and DV. All authors reviewed the manuscript and gave final approval for the work.

## Conflict of Interest Statement

The authors declare that the research was conducted in the absence of any commercial or financial relationships that could be construed as a potential conflict of interest.

## Supplementary Material

Figure S1 B cell phenotype of granulocyte monocyte colony-stimulating factor (GM-CSF)+ and − cells from disease controls. Freshly isolated peripheral blood mononuclear cells from eight disease controls (osteoarthritis = 4 and psoriatic arthritis *n* = 4) were stimulated and stained as described in Section “Materials and Methods” and in Figure 2. The % of each B cell subpopulation was compared between GM-CSF+ (black bars) and − (white bars) cells. The bars represent the mean ± SD (%) of each subtype. The *p*-values from the comparison between the two groups are also shown.

Figure S2 Granulocyte monocyte colony-stimulating factor (GM-CSF)+ B or T cells in rheumatoid arthritis (RA) patients according to their serological status [rheumatoid factor (RF)/anticyclic citrullinated peptide (anti-CCP)]. The % expression of GM-CSF+ B (CD19+) or T (CD3+) cells in RF and/or anti-CCP positive (*n* = 20) or negative (*n* = 20) RA patients is shown. There was no statistically significant difference between the two groups either for B (*p* = 0.250) or T (*p* = 0.892) cells.
