# Supplementary figures and images for: Increased Frequency of Peripheral B and T Cells Expressing Granulocyte Monocyte Colony-Stimulating Factor in Rheumatoid Arthritis Patients
Source: Front Immunol. 2018 Jan 10;8:1967. doi: 10.3389/fimmu.2017.01967 (PMC5767588; doi:10.3389/fimmu.2017.01967)

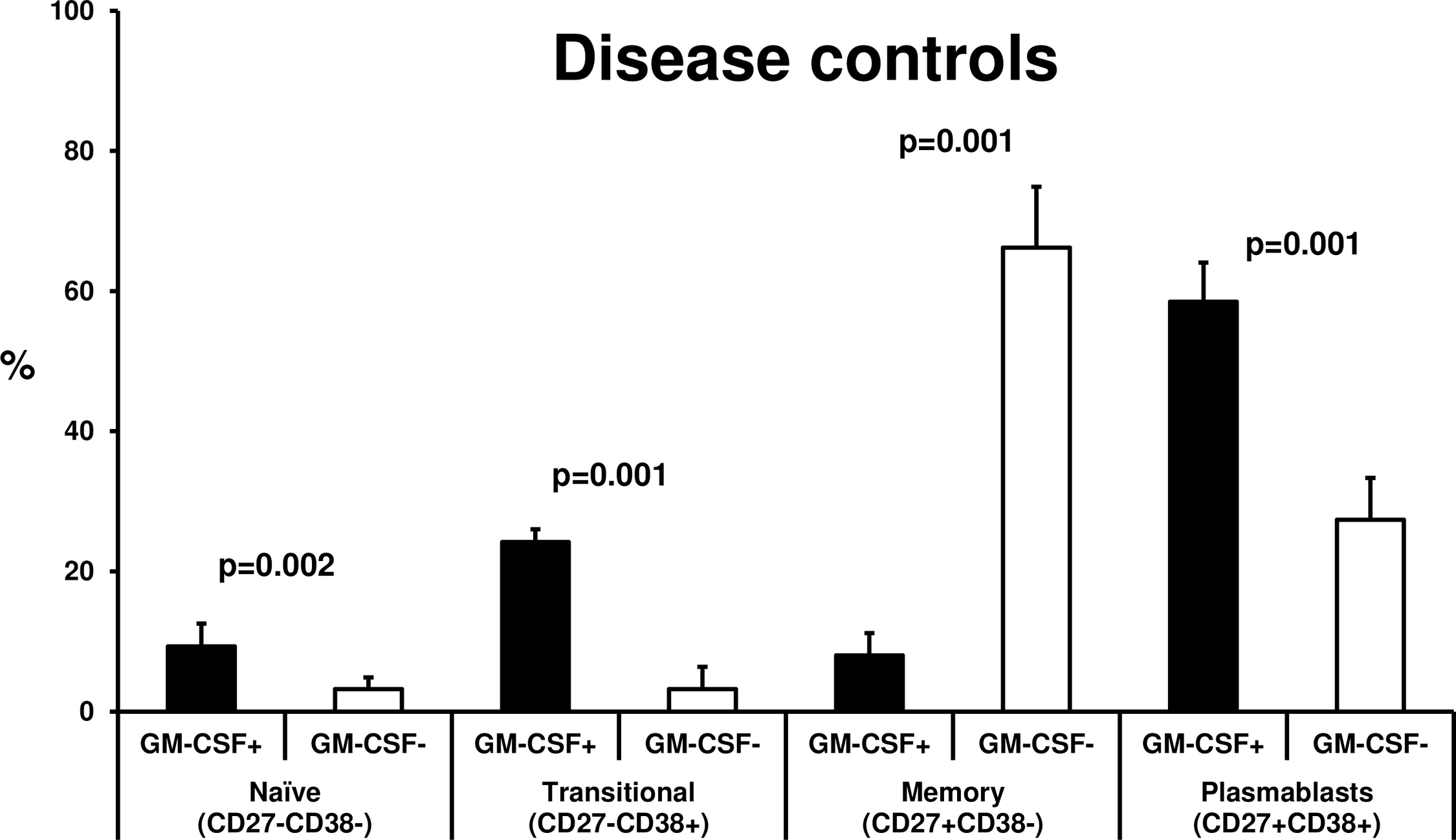

Supplement: Figure S1 — B cell phenotype of granulocyte monocyte colony-stimulating factor (GM-CSF)+ and − cells from disease controls. Freshly isolated peripheral blood mononuclear cells from eight disease controls (osteoarthritis = 4 and psoriatic arthritis n = 4) were stimulated and stained as described in Section “Materials and Methods” and in Figure 2. The % of each B cell subpopulation was compared between GM-CSF+ (black bars) and − (white bars) cells. The bars represent the mean ± SD (%) of each subtype. The p-values from the comparison between the two groups are also shown. [file image_1.tif]

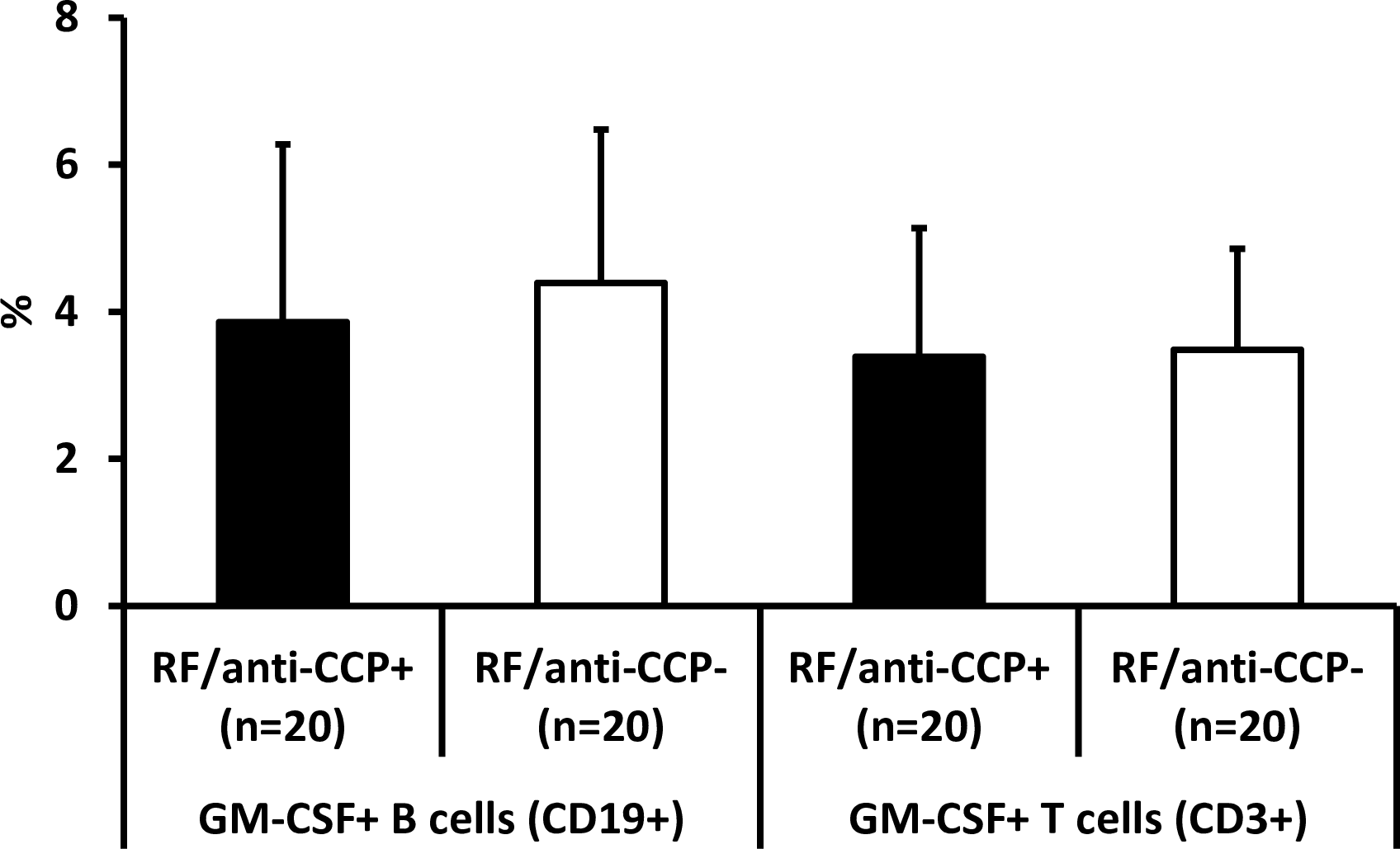

Supplement: Figure S2 — Granulocyte monocyte colony-stimulating factor (GM-CSF)+ B or T cells in rheumatoid arthritis (RA) patients according to their serological status [rheumatoid factor (RF)/anticyclic citrullinated peptide (anti-CCP)]. The % expression of GM-CSF+ B (CD19+) or T (CD3+) cells in RF and/or anti-CCP positive (n = 20) or negative (n = 20) RA patients is shown. There was no statistically significant difference between the two groups either for B (p = 0.250) or T (p = 0.892) cells. [file image_2.tif]
